# High intraspecies allelic diversity in *Arabidopsis* NLR immune receptors is associated with higher transcription, gene body hypomethylation, and proximity to transposable elements

**DOI:** 10.1101/2023.01.12.523861

**Published:** 2023-01-13

**Authors:** Chandler A. Sutherland, Daniil M. Prigozhin, J. Grey Monroe, Ksenia V. Krasileva

**Affiliations:** 1 Department of Plant and Microbial Biology, University of California Berkeley, Berkeley, CA, USA 94720; 2 Molecular Biophysics and Integrated Bioimaging Division, Lawrence Berkeley National Laboratory, Berkeley, CA, USA 94720; 3 Department of Plant Sciences, University of California Davis, Davis, CA, USA 95616

## Abstract

Plants rely on Nucleotide-binding, Leucine-rich repeat Receptors (NLRs) for pathogen recognition. Highly variable NLRs (hvNLRs) show remarkable intra-species diversity, while their low variability paralogs (non-hvNLRs) are conserved between ecotypes. At a population level, hvNLRs provide new pathogen recognition specificities, but the association between allelic diversity and genomic and epigenomic features has not been established. Our investigation of NLRs in *Arabidopsis* Col-0 has revealed that hvNLRs show higher expression, less gene body cytosine methylation, and closer proximity to transposable elements than non-hvNLRs. How these features are established, maintained, and potentially driving the difference in the observed diversity of hv and non-hvNLRs is key to understanding the evolution of plant innate immune receptors.

## Introduction

Plants, lacking the adaptive immune systems of vertebrates, use germline-encoded innate immune receptors to defend against rapidly evolving pathogens. Despite their inability to create antibodies through hypermutation and recombination, plants remain quite successful against pathogens due to remarkable receptor diversity at the population level. Nucleotide-binding, Leucine-rich repeat Receptors (NLRs) are the intracellular sensors of the plant immune system, detecting pathogen-secreted, disease-promoting effector proteins. NLRs initiate defense responses through oligomerization of the central nucleotide-binding domain, initiating transcriptional reprogramming, hormone induction, and hypersensitive cell death response (Ngou, Ding, and Jones 2022). Plant NLRs are differentiated into three anciently diverged classes based on their N-terminal domains: Resistance To Powdery Mildew 8-NLR (RNL), Coiled-Coil-NLR (CNL), or Toll/Interleukin-1 Receptor-NLR (TNL).

Among individuals of a given species, NLRs vary greatly in sequence diversity (Barragan and Weigel 2021). Systematic analysis of pan-NLRomes from 62 *Arabidopsis thaliana* accessions, and 54 *Brachypodium distachyon* lines found that highly variable NLRs (hvNLRs) are distributed across the NLR phylogeny and are interspersed with low-variability paralogs (non-hvNLRs) (Prigozhin and Krasileva 2021). At the population level, hvNLRs are hypothesized to act as reservoirs of diversity for future pathogen effectors, while non-hvNLRs may retain successful binding sequences. These observations have raised questions about whether elevated mutation rates play a role in generating hvNLR diversity.

Mutation rates are unlikely to evolve on a gene by gene basis in response to selection given the barrier imposed by genetic drift (Lynch 2010). However, selection on genic mutation rates is sufficiently strong when acting on mechanisms that couple mutation rate to expression states and epigenomic features, affecting the mutation rates of many genes simultaneously (Martincorena and Luscombe 2013). The mutation rate of *Arabidopsis* is heterogeneous across the genome, consistent with expected effects of selection on mechanisms linking mutation rates to epigenomic features (Monroe et al. 2022). Therefore, investigation of differences between the epigenomic, sequence, and regulatory features (hereafter genomic features) of hv and non-hvNLRs may lead to mechanistic explanations of their enhanced allelic diversity. In this paper, we report that hvNLRs show a higher transcription level, closer proximity to transposable elements (TEs), and less gene body CG cytosine methylation than non-hvNLRs. These findings will serve as a starting point for the investigation of the mechanisms that promote diversity generation in a subset of plant immune receptors.

## Results

**Figure 1:**
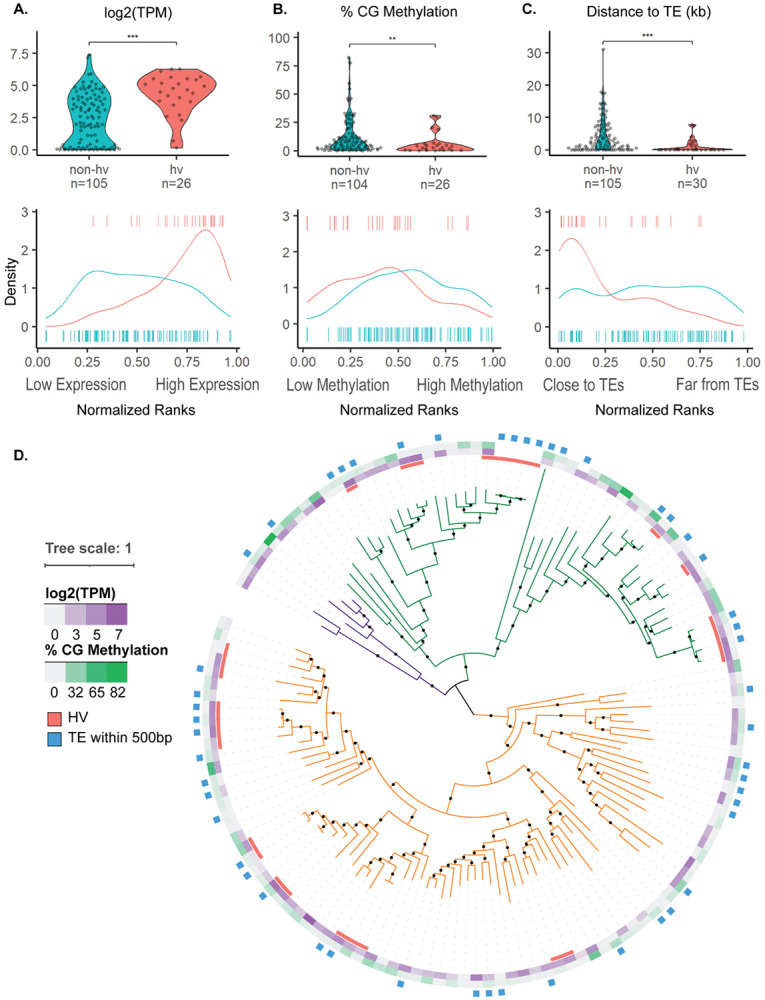
Expression, methylation, and proximity to transposable elements distinguish hv and non-hv NLRs. **A:** average gene expression log2 (Transcripts per Million), **B:** average % CG methylation per gene, and **C:** distance to nearest transposable element (kbp) with normalized mean percentile rank density plots of hv and non-hvNLRs.* indicates a p-value between<0.05 and >= 0.01; ** indicates a p-value between 0<.01 and >=0.001; *** indicates a p-value <0.001. **D:** Features mapped onto a phylogeny of NLRs in *A. thaliana* Col-0. RNL, CNL, and TNL clades are colored in purple, green, and orange, respectively. NLRs without log2(TPM) or % CG methylation data were determined to be unmappable (see methods).

To compare the expression and methylation status of hv and non-hvNLRs within an individual plant, we examined available matched whole genome bisfulfite and RNA sequencing data from the rosette leaf tissue of four *A. thaliana* Col-0 plants without pathogen exposure (Williams et al. 2022). hvNLRs are more highly expressed than non-hvNLRs (Fig. 1A, t-test, p=2.6e-06), and hvNLRs are enriched in the high abundance transcripts in each leaf sample (singscore rank-based sample scoring, hvNLR p < 0.01 for each biological replicate) (Foroutan et al. 2018).

In addition, hvNLRs have lower gene body CG methylation than non-hvNLRs (Fig. 1B, t-test, p=0.0068), and hvNLRs are enriched in the CG hypomethylated genes across the genome (Fig. 1B, permutation test for difference in means, p = 0.02, n=10,000 replicates). Gene set analysis of methylation data is known to be biased due to the uneven distribution of CG sites within each gene (Geeleher et al. 2013). To address this, we repeated our permutation test to compare hvNLRs to a set of non-NLR genes with similar measured CG sites per gene, and the result was still significant for hvNLRs in three of four biological replicates (p < 0.05 for three biological replicates, p=0.07 for the fourth biological replicate, n=10,000 replicates). CHH and CHG context methylation is not typically found in the gene body unless there is a nearby or overlapping TE insertion. There was no difference in gene body CHH and CHG context methylation between hv and non-hvNLRs.

TEs are major drivers of genome evolution, and mutagenic in their insertions and excisions. hvNLRs are much more likely to be near TEs (Fig. 1C, t-test, p = 1.7e-06), and hvNLRs are enriched in the genes closest to TEs (permutation test for difference in medians, n=10,000 replicates, p=0 hvNLRs). In Col-0, hvNLRs have a median TE distance of 0 kbp, meaning the TEs are within the UTR or intronic sequences, while non-hvNLRs have a median TE distance of 2.07 kbp. Highly variable status is predictive of TEs within the genic sequence (Chi-squared test, p=0.0007). We concluded that hvNLRs are much more likely to be near or overlapping with TEs than non-hvNLRs.

**Figure 2:**
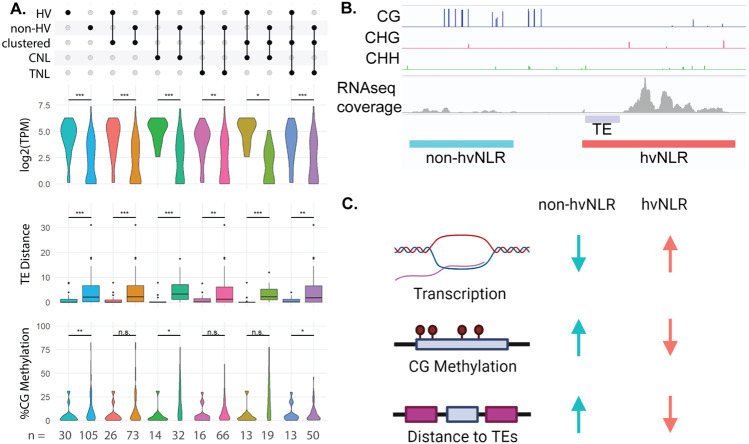
Cluster membership and NLR type do not exclusively account for genomic differences between hv and non-hvNLRs. **A:** Comparison of expression, CG gene body methylation, and distance to nearest TEs of hv and non-hvNLRs by cluster membership and N-term domain type. **B:** Methylation and RNAseq coverage of neighboring non-hvNLR AT5G43725 and hvNLR AT5G43740. **C:** Summary of presented results.

NLRs are found in clusters more frequently than other genes (Lee and Chae 2020), with variable membership of hv and non-hvNLRs. However, hv and non-hvNLRs maintain their distinct expression and TE-association patterns when comparing exclusively clustered hv and non-hvNLRs and within the CNL and TNL N-terminal domain clades (Fig. 1D; 2A). CG methylation, however, is not significantly different between clustered hv and non-hvNLRs and between TNLs (Fig. 2A). CG methylation is the weakest association with hv status of the three examined features (Fig 1B), and further analysis with more accessions will reveal if cluster or hv status is more predictive of CG methylation. hvNLRs are distributed over the phylogeny of NLRs (Fig 1D), but despite close phylogenetic relationships with non-hvNLRs, maintain distinct genomic features. Here, we show that the methylation status, expression, and TE distance in Col-0 are associated with intraspecies allelic diversity of hvNLRs.

## Discussion

Our results show that differences in NLR allelic diversity are correlated with certain genomic features. hvNLRs are more expressed than non-hvNLRs, and enriched across the genome in highly expressed genes. Transcription is a source of genomic instability through the exposure of vulnerable single-stranded DNA, but can also target DNA repair machinery to actively transcribed genes (Oztas et al. 2018). If expression is related to increased mutation rate in *Arabidopsis*, this could contribute to the rapid diversification of hvNLRs. We found that non-hvNLRs are more methylated than hvNLRs. Methylated cytosines are positively correlated with mutation rate due to the increased frequency of spontaneous deamination of cytosines (Monroe et al. 2022; Alexandrov et al. 2020). However, in *Arabidopsis*, gene body CG methylation is preferentially in the exons of conserved, constitutively transcribed housekeeping genes, though any conferred benefit is unknown (Gaut et al. 2011; He et al. 2022). The gene body methylation of non-hvNLRs may be a mechanism for sequestering successful receptors through some undetermined mechanism, despite the increased risk of mutation. TEs mutagenic in their insertions and excisions, and alter the methylation and expression landscape of surrounding genes. hvNLRs are closer to TEs and more likely to have them within their genic sequence than non-hvNLRs, and this likely contributes to hvNLR diversification. It has been previously observed that TEs are associated with plant immune genes (Kawakatsu et al. 2016), but this analysis suggests that signal may be driven by hvNLRs (Figure 1C; Hosaka and Kakutani 2018).

Evolution of innate immune receptors is a rare case in which a high mutation rate may be beneficial. Given the heterogeneous mutation rate across the *Arabidopsis* genome, it is tempting to speculate that distinctive genomic features we observed in hv and non-hvNLRs may be driving their allelic diversity. Our findings serve as a starting point for the investigation of the mechanisms that promote diversity generation in a subset of the plant immune receptors.

## Materials and Methods

To examine the methylation and expression of NLRs, we used available matched bisulfite and RNA sequencing from split Col-0 leaves (Williams et al. 2022). Reads were trimmed using Trim Galore! v0.6.6 with a Phred score cutoff of 20 and Illumina adapter sequences, with a maximum trimming error rate 0.1 (Babraham Bioinformatics). Using Bismark v0.23.0, reads were mapped to the Araport11 genome, PCR duplicates were removed, and percent methylation at each cytosine was determined using the methylation extraction function (Krueger and Andrews 2011). Cytosines with at least 5 reads were used for analysis, and the symmetrical cytosines within CG base pairs were averaged (Williams et al. 2022). The percent methylation of each CG site was averaged across each NLR gene, and across four biological replicates. Five hvNLR genes did not have sufficient coverage at any cytosines and were excluded from analysis (AT1G58807, AT1G58848, AT1G59124, AT1G59218, and AT4G26090).

RNA-seq reads from four matched leaf samples (explained above) were mapped to the Araport11 genome using STAR v2.7.10a and were counted using htseq-count v2.0.2 (Dobin et al. 2013). Counts were converted to transcripts per million and averaged across four biological replicates, then log2 transformed for visualization. NLRs are repetitive and often similar, making them difficult to sequence with short reads. To determine if any NLRs were unmappable, RNAseq reads were simulated using Polyester v1.2.0 (Frazee et al. 2015). Four NLRs were determined to be unmappable due to zero assigned read counts and were excluded from expression analysis (AT1G58807, AT1G58848, AT1G59124, and AT1G59218). Single sample gene set enrichment of hvNLRs and non-hvNLRs was performed on each replicate using singscore (Foroutan et al. 2018).

We determined distance to transposable elements based on TE annotation file TAIR10_Transposable_Elements.txt and gene annotation file TAIR10_GFF3_genes.gff available from arabidopsis.org. The phylogenetic tree of all NLRs in Col-0 was generated as described previously (Prigozhin and Krasileva 2021) with feature annotations using iTOL. The UpSet plot was generated using the R package ComplexUpset v1.3.3.

## Data Availability

All the data generated in this study is hosted on the Zenodo Public Repository at 10.5281/zenodo.7527905. The processing pipelines and figure generation code are available on Github (https://github.com/chandlersutherland/nlr_features).
