# Supplementary material for: High intraspecies allelic diversity in Arabidopsis NLR immune receptors is associated with distinct genomic and epigenomic features
Source: bioRxiv. 2023 Oct 5:2023.01.12.523861. Originally published 2023 Jan 13. Preprint. [Version 2] doi: 10.1101/2023.01.12.523861 (PMC9882162; doi:10.1101/2023.01.12.523861)
Supplement: 1 [file NIHPP2023.01.12.523861V2-supplement-1.pdf]

bioRxiv preprint doi: https://doi.org/10.1101/2023.01.12.523861; this version posted October 5, 2023. The copyright holder for this preprint (which was not certified by peer review) is the author/funder, who has granted bioRxiv a license to display the preprint in perpetuity. It is made available under aCC-BY 4.0 International license.

Supplemental Figures

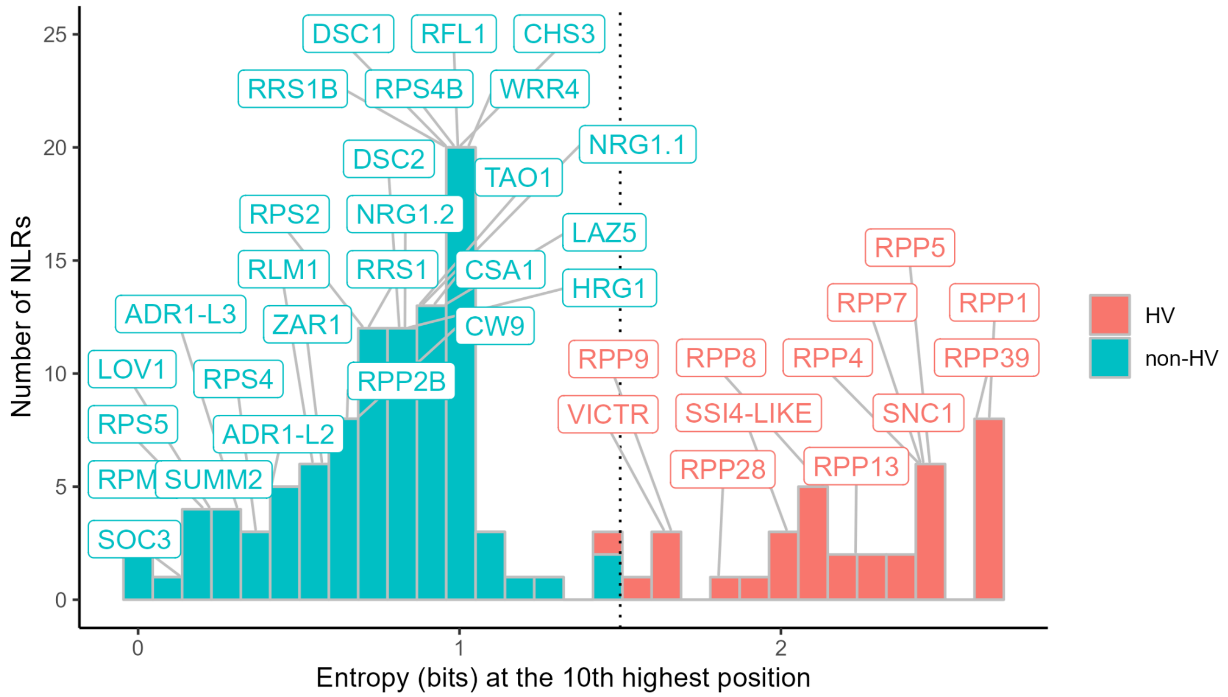

Supplemental Figure 1: Distribution of NLR Shannon entropy at the tenth highest amino acid position as shown as a histogram with 30 bins. Described NLRs are annotated. The designation of hvNLR is entropy of >1.5 bits at the tenth highest position, as shown by the dashed line.

bioRxiv preprint doi: https://doi.org/10.1101/2023.01.12.523861; this version posted October 5, 2023. The copyright holder for this preprint (which was not certified by peer review) is the author/funder, who has granted bioRxiv a license to display the preprint in perpetuity. It is made available under aCC-BY 4.0 International license.

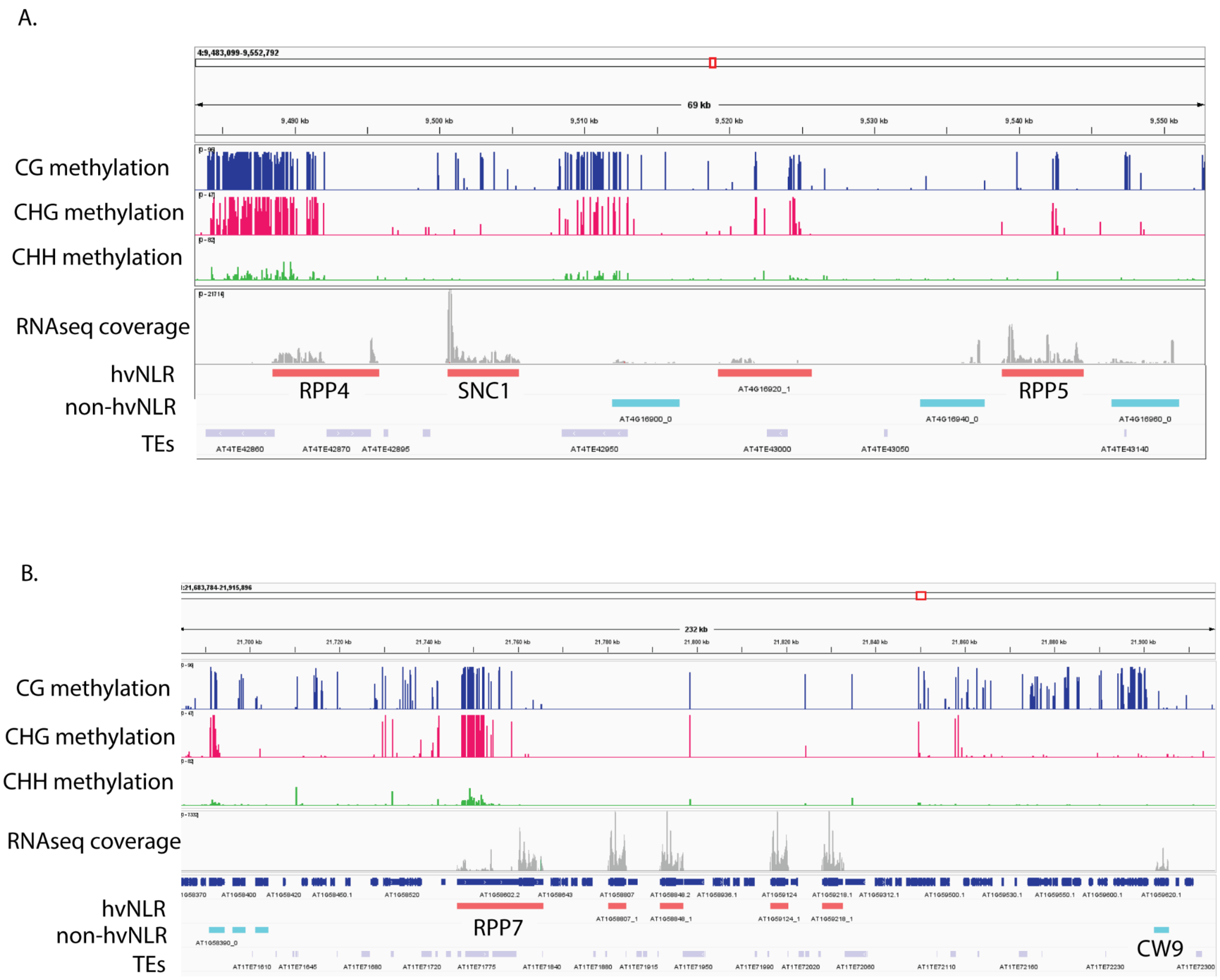

Supplemental Figure 2: Integrative Genomics Viewer screenshot of Methylation, RNAseq coverage, and TE proximity of the **A.** *RPP4* and **B.** *RPP7* clusters.

bioRxiv preprint doi: https://doi.org/10.1101/2023.01.12.523861; this version posted October 5, 2023. The copyright holder for this preprint (which was not certified by peer review) is the author/funder, who has granted bioRxiv a license to display the preprint in perpetuity. It is made available under aCC-BY 4.0 International license.

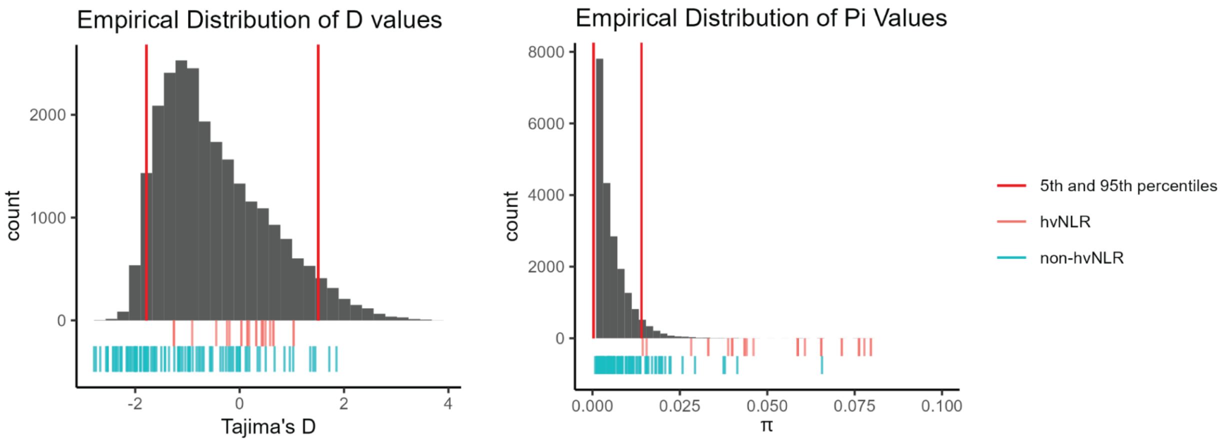

Supplemental Figure 3: Empirical distribution of Tajima's D and π calculated on coding sequences of *Arabidopsis*. Position of hv and non-hvNLRs shown via rug plot, as well as the 5th and 95th percentiles of the distribution.

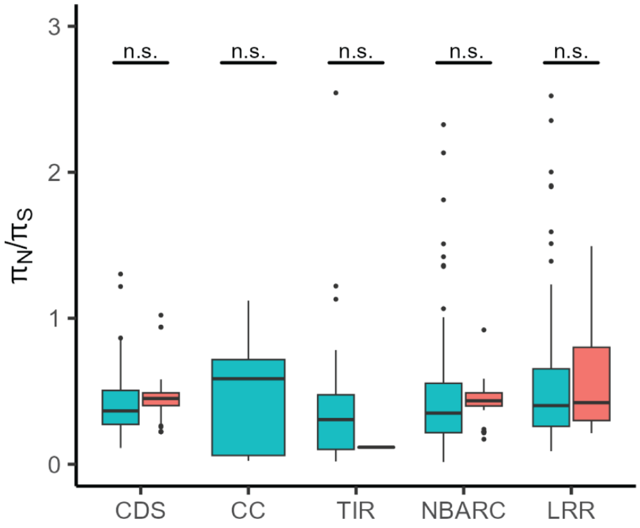

Supplemental Figure 4: π_N/π_S calculated per domain.
